# Improving diffuse optical tomography imaging quality using APU-Net: an attention-based physical U-Net model

**DOI:** 10.1117/1.JBO.29.8.086001

**Published:** 2024-07-25

**Authors:** Minghao Xue, Shuying Li, Quing Zhu

**Affiliations:** a Washington University in St. Louis, Biomedical Engineering Department, St. Louis, Missouri, United States; b Boston University, Electrical and Computer Engineering Department, Boston, Massachusetts, United States; c Washington University in St. Louis, Radiology Department, St. Louis, Missouri, United States

**Keywords:** diffuse optical tomography, ultrasound, image enhancement, deep learning, attention-based U-Net

## Abstract

**Significance:**

Traditional diffuse optical tomography (DOT) reconstructions are hampered by image artifacts arising from factors such as DOT sources being closer to shallow lesions, poor optode-tissue coupling, tissue heterogeneity, and large high-contrast lesions lacking information in deeper regions (known as shadowing effect). Addressing these challenges is crucial for improving the quality of DOT images and obtaining robust lesion diagnosis.

**Aim:**

We address the limitations of current DOT imaging reconstruction by introducing an attention-based U-Net (APU-Net) model to enhance the image quality of DOT reconstruction, ultimately improving lesion diagnostic accuracy.

**Approach:**

We designed an APU-Net model incorporating a contextual transformer attention module to enhance DOT reconstruction. The model was trained on simulation and phantom data, focusing on challenges such as artifact-induced distortions and lesion-shadowing effects. The model was then evaluated by the clinical data.

**Results:**

Transitioning from simulation and phantom data to clinical patients’ data, our APU-Net model effectively reduced artifacts with an average artifact contrast decrease of 26.83% and improved image quality. In addition, statistical analyses revealed significant contrast improvements in depth profile with an average contrast increase of 20.28% and 45.31% for the second and third target layers, respectively. These results highlighted the efficacy of our approach in breast cancer diagnosis.

**Conclusions:**

The APU-Net model improves the image quality of DOT reconstruction by reducing DOT image artifacts and improving the target depth profile.

## 1 Introduction

In the United States, breast cancer is the most diagnosed and the second deadliest cancer. With an estimated 297,790 new cases and 43,170 deaths annually, it continues to be a significant public health concern, according to the American Cancer Society.^1^

X-ray mammography, magnetic resonance imaging (MRI), and ultrasound (US) have been applied in cancer screening and detection.^2^^–^^14^ However, mammography is known to exhibit low contrast and sensitivity, particularly in younger women with dense breasts.^10^^–^^13^ MRI, on the other hand, is constrained by the requirement for contrast agent injection, and the diagnostic utility of US for solid masses is also limited.

To address these limitations, our group developed a portable frequency domain US-guided diffuse optical tomography (DOT) system.^15^^,^^16^ DOT utilizes scattered near-infrared light to reconstruct the distributions of optical absorption coefficients at selected wavelengths and map the hemoglobin concentration of the biological tissue.^17^^,^^18^ Researchers have extensively investigated the use of DOT in the diagnosis of breast cancer and the estimation of tissue optical properties.^19^^–^^24^ By incorporating DOT into breast cancer diagnosis, we can potentially improve the accuracy of breast cancer detection and reduce the need for unnecessary biopsies, ultimately improving patient outcomes.

**Fig. 1:**
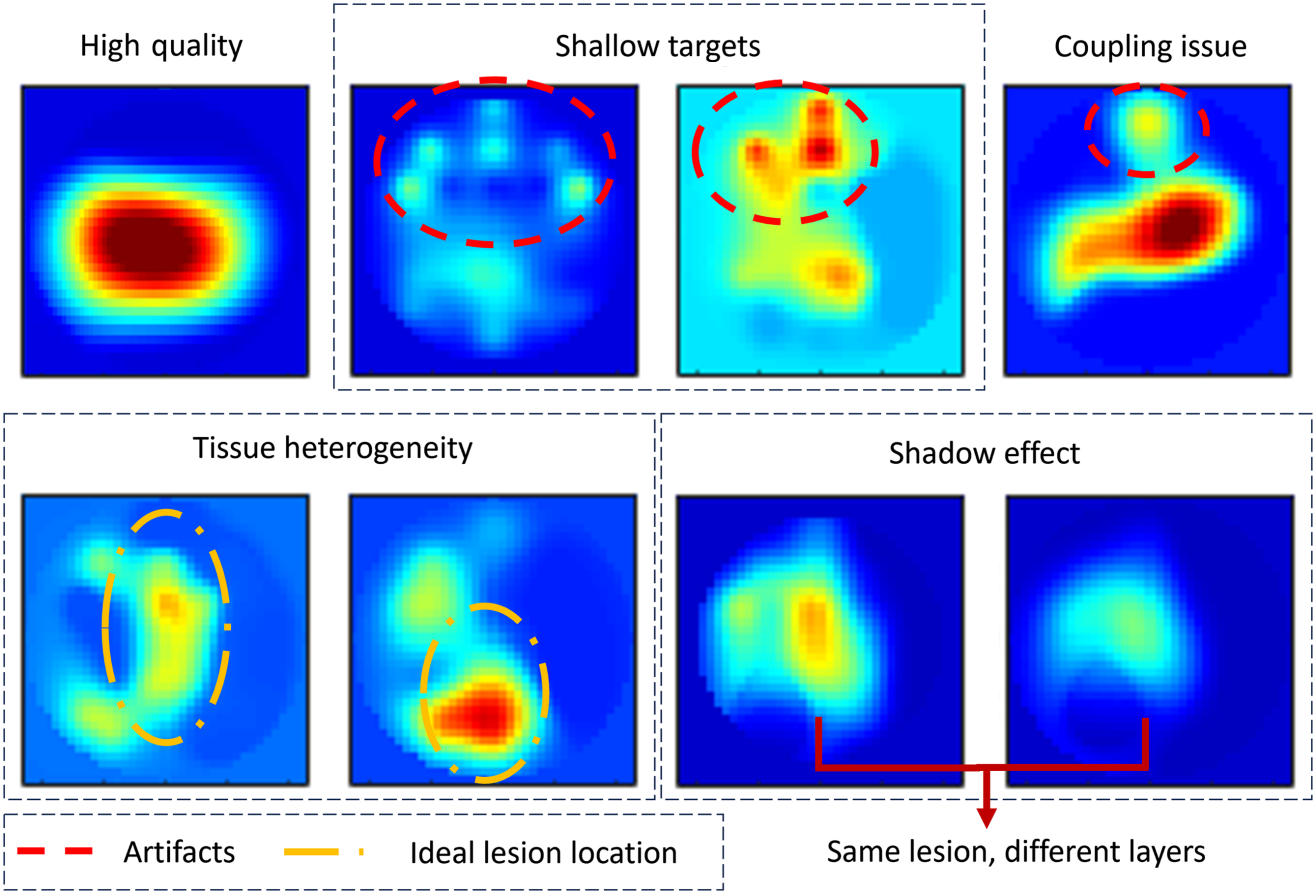
Degraded DOT reconstructions. By rows, from left to right: high-quality reconstruction, reconstruction of shallow targets with source distribution on top, reconstruction with optode coupling issue, reconstruction with multiple objects due to tissue heterogeneity, and the shadow effect caused by the absorption of the top layer.

DOT has been proven to downgrade the biopsy recommendation of Breast Imaging Reporting and Data System assessments by 23.5% for benign lesions,^25^ implying a huge potential for reducing high false positives in US-based diagnoses. However, as illustrated in Fig. 1, the quality of DOT images is degraded by many problems: source artifacts when imaging shallow lesions, artifacts caused by poor optode–tissue coupling, a mismatch between the reference and target sides, tissue heterogeneity, and lesion posterior shadowing. The reconstruction of shallow targets, which are located close to the probe-tissue interface, tends to include sources on the probe itself that impede getting a correct hemoglobin reading from the images. DOT reconstructions are also sensitive to measurement errors when the probe is in poor contact with the tissue and tissue heterogeneity. The former problem causes hot spots on the non-lesion regions, for example, at the edge of the reconstructed DOT image, while the latter issue introduces multiple target-like objects. Besides, a large, high-absorption lesion absorbs more light from the top layer in depth and causes fewer photons to penetrate deeper layers; thus, the reconstructed absorption profile loses the details in the deeper region. Therefore, improving the quality of DOT reconstruction is essential to downstream tasks, including diagnosing malignant and benign lesions.

Recently, many research groups have proposed various deep learning-based methods for DOT reconstruction and quality improvement. Zhao et al.^26^ introduced an unroll-DOT framework, utilizing a refined U-Net to enhance DOT images following an unroll-network process. Ko et al.^27^ integrated deep neural networks with conventional DOT reconstruction methods, resulting in a notable enhancement in image quality compared with traditional approaches. Yedder et al.^28^ presented a multitask deep learning framework for reconstruction and lesion localization in limited-angle DOT. They used physics-based simulations to create synthetic datasets and applied a transfer learning approach to bridge the sensor domain gap between *in silico* and real-world data, yielding promising results in a clinical example. Deng et al.^24^ developed the FDU-Net, which consists of a fully connected subnet, a convolutional encoder-decoder subnet, and a U-Net, for three-dimensional DOT reconstruction, also demonstrating favorable outcomes in one clinical case. However, these approaches have limitations. Many of them lack extensive validation with clinical datasets or have been tested on only one or two patient cases. The challenges inherent in DOT reconstruction, compounded by image resolution limitations, hinder the widespread adoption of deep learning techniques in DOT image enhancement. Besides, the absence of ground truth in clinical images introduces a significant barrier to supervised learning due to the domain shift issues between the simulated and clinical data.

Other than the artifacts of DOT images, one primary challenge in reconstruction is the impact of target size and depth, which can adversely affect the accuracy of the reconstructed absorption maps. Specifically, smaller and deeper targets are often under-reconstructed, which means the reconstructed lesion suffers from a lower absorption coefficient, limiting the diagnostic accuracy.

U-Net has emerged as a pivotal architecture in image enhancement due to its unique ability to preserve spatial information while effectively capturing contextual features. U-Net–based models have demonstrated versatility and effectiveness in image reconstruction applications,^29^^–^^32^ such as those by Chen et al.^30^ and Chowdary and Yin.^31^ The skip connections in U-Net enable the precise reconstruction of images, making it particularly useful for tasks where accurate localization and reconstruction are required. Given these strengths, our use of a U-Net–based model is aimed at achieving high-fidelity reconstructions with improved accuracy, building on its proven track record in image processing tasks. This design helps address the challenges posed by variable target sizes and depths, leading to improved accuracy.

However, U-Net’s performance may be affected by the low resolution of functional DOT inputs. Thus, we introduced the contextual transformer (CoT) attention module^33^ into the U-Net to obtain a more target-focused and highly generalizable deep learning model. The attention module, a pivotal component in modern neural network architectures, facilitates the dynamic weighting of input features, allowing the model to selectively focus on relevant information. By applying the attention module, the model focused more on the relationship between the adjacent depth layers of the DOT reconstruction and the artifacts around the target in the image.

In this study, we present a novel deep learning framework with an attention module to enhance the contrast and remove the artifacts in DOT reconstruction. The attention-based U-Net (APU-Net) model takes the reconstructed DOT image and the corresponding fine mesh information as the input, predicting the measurement in the bottleneck as the forward model, and then outputs the enhanced DOT images as the solver of the inverse problem for DOT reconstruction. After training exclusively on simulation and phantom data, the model demonstrated commendable efficacy in enhancing depth contrast and eliminating artifacts in clinical DOT images. To our knowledge, this is the first application of a deep learning model to enhance DOT reconstructions with a large patient dataset, with potential applicability to other DOT systems.

## 2 System Structure and Methods

### 2.1 System Structure

A frequency-domain US-guided DOT system designed by our group was utilized to collect phantom and patient data.^16^ The system employed a hand-held probe that integrated nine source fibers and 14 detection fibers. The light was delivered sequentially at four wavelengths (730, 785, 808, and 830 nm) to each of the nine fibers, and 14 parallel photomultiplier tube detectors detected the light from each source position simultaneously. The laser diodes were modulated at 140.02 MHz, and the system utilized heterodyne detection to mix the detected signals with a 140-MHz reference signal to generate 20-kHz signals. Following this, the output of the mixer for each channel underwent amplification and filtering at 20 kHz before being sampled by a 16-channel analog-to-digital converter.

**Fig. 2:**
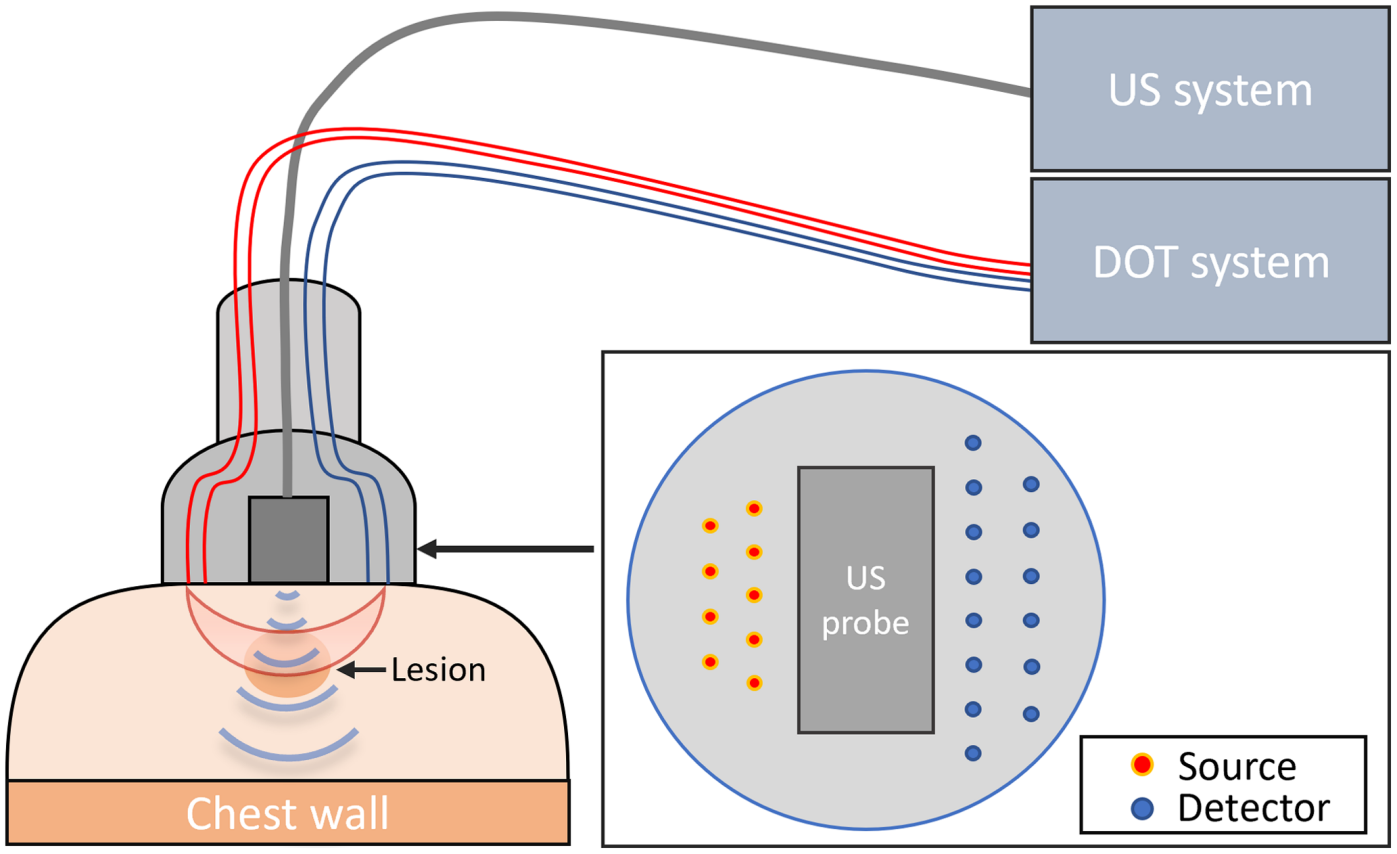
Sketch of the DOT system. The probe is placed on the compressed breast.

For real-time US image guidance, we positioned a commercial linear US probe at the center of the DOT probe to obtain US and DOT measurements of the targeted lesion beneath, as detailed in Ref. 34. The system is depicted in Fig. 2. The probe position was fixed during DOT data acquisition, with a data acquisition time of ∼3 to 4 s for each data set. US recording was paused during DOT data acquisition and resumed once the DOT data collection was complete.

### 2.2 Simulation and Phantom Configuration

To mimic the clinical dataset as much as possible and make our model more generalizable to the clinical dataset, we employed the finite element method (FEM) in COMSOL software and conducted Monte Carlo (MC) simulations using the Virtual Imaging Clinical Trial for Regulatory Evaluation (VICTRE)^35^ breast phantom to generate forward measurements. The FEM simulation can replicate the DOT reconstruction with artifacts in shallow targets. Meanwhile, the MC simulation sought to reproduce DOT reconstruction with artifacts related to tissue heterogeneity by varying the fat fraction within the digital breast phantom.

For the FEM approach, we approximated complex clinical scenarios as a single-target breast-shaped model. In the setting of geometry, a hemisphere with a radius of 10 cm was employed. Various inclusions, such as a ball, ellipsoid, cube, a ball with two combined hemispheres, a star, and letters, were considered with varying absorption coefficients to improve the complexity of the dataset. In the simulation, we positioned nine sources and 14 detectors based on the geometry of our clinical DOT probe. Further details of this setting can be found in Ref. 36.

In the MC approach, the digital phantom generated by VICTRE, with a radius of 7 cm and height of 5 cm, served as a heterogeneous condition by incorporating various tissue types. Simulations were performed by translating and rotating the probe on the phantom, with fat fractions varying from 20% to 80%. Additional details of the MC simulation can be found in Ref. 37.

For the phantom study, we utilized high-contrast targets made of calibrated polyester resin ($\mu_{a}=0.23\;{\mathrm{cm}}^{-1}$) and low-contrast targets made of silicon ($\mu_{a}=0.11\;{\mathrm{cm}}^{-1}$). The targets were immersed in an intralipid solution ($\mu_{a}=0.02\;{\mathrm{cm}}^{-1}$, $\mu_{s}^{\prime }=6-8\;{\mathrm{cm}}^{-1}$) and placed over a silicon plate whose $\mu_{a}$ was similar to that of the solution. We recorded the co-registered US images and the DOT measurements with different targets centralized underneath the probe.

In this study, we utilized 10,975 sets of simulation data and 360 sets of phantom data. Further dataset details are provided in Table 1.

**Table 1:** Range of parameters used in simulations and phantoms.

	Simulation		Phantom
	FEM	MC	
Target size (diameter/length)/cm	∼1.0 to 4.0	∼1.0 to 3.0	1.0 to 3.0
Target center depth/cm	∼0.8 to 3.5	∼1.5 to 2.5	1.0 to 3.5
Target $\mu_{a}$ ${\mathrm{cm}}^{-1}$	∼0.1 to 0.3	∼0.1 to 0.2	0.11/0.23
Target $\mu_{s}^{\prime }$ ${\mathrm{cm}}^{-1}$	∼4.0 to 8.0	∼4.0 to 8.0	6.0
Background tissue $\mu_{a}$ ${\mathrm{cm}}^{-1}$	∼0.02 to 0.06	Fat fraction ∼20% to 80%	0.02
Background tissue $\mu_{s}^{\prime }$ ${\mathrm{cm}}^{-1}$	∼4.0 to 8.0	Fat fraction ∼20 to 80%	6.0
Chest wall $\mu_{a}$ ${\mathrm{cm}}^{-1}$	∼0.1 to 0.2	—	—

### 2.3 DOT Patient Data

Our US-guided DOT system has been utilized in clinical studies with protocols that received approval from the appropriate Institutional Review Boards and complied with the Health Insurance Portability and Accountability Act.^38^^,^^39^ All participants were fully informed about the study’s purpose, procedures, and potential risks before signing a written consent form. To maintain patient confidentiality, all data used in this study were de-identified. Table 2 lists the details of the histologic group, age, histologic diagnosis, and information on shallow cases.

**Table 2:** Patient information.

Histologic group (n)	Age (years) (range)	Histologic diagnosis on biopsy (n)	Shallow cases with artifacts (n) (average age)
Benign (53)	43.25±12.00 (18−72)	Fibrocystic changes (22)	26 (40.25±12.28)
		Fibroadenomatous (24)	
		Proliferative (7)	
Malignant (30)	57.29±13.72 (34−81)	Invasive ductal carcinoma (18)	8 (64.71±15.28)
		Invasive lobular carcinoma (3)	
		Invasive mucinous carcinoma (4)	
		Invasive mammary carcinoma (1)	
		Papillary carcinoma (4)	

### 2.4 Conjugate Gradient Descent (CGD) Reconstruction

We used the CGD reconstruction as the input for our model. Details about this reconstruction method can be found in Ref. 40. In summary, we modeled photon migration using a diffusion equation for the photon density wave and applied the Born approximation to relate the scattered field ($U_{\mathrm{sc}}$) to the changes in absorption coefficients ($\delta \mu_{a}$), as follows:

$${\left[U_{\mathrm{sc}}\right]}_{m\times 1}={\left[W\right]}_{m\times n}{\left[\delta \mu_{a}\right]}_{n\times 1},$$ (1)

where W is the weight matrix derived from the diffusion equation for a semi-infinite medium. The variable m represents the number of measurements, and n represents the number of voxels.

To solve for $\delta \mu_{a}$, we formulated the inverse problem as

$${\arg\;\min}_{\delta \mu_{a}}({\left\|U_{sc}-W\delta \mu_{a}\right\|}^{2}+\frac{\lambda }{2}{\left\|\delta \mu_{a}-\delta \mu_{a}^{0}\right\|}^{2}),$$ (2)

where ‖·‖ is the Euclidean norm, $\delta \mu_{a}^{0}$ is the initial estimate of the optical properties, and λ is the regularization parameter. This formulation allows us to estimate the changes in absorption coefficients while balancing the data-fitting term and the regularization term, which contributes to the stability and accuracy of the reconstruction.

The spatial grid used for reconstruction measures 9  cm×9  cm×3.5  cm. This grid is divided into a fine-mesh grid centered at the lesion location and a coarse-mesh grid for the background. The resolutions for the fine and coarse meshes are 0.25  cm×0.25  cm×0.5  cm and 1.5  cm×1.5  cm×0.5  cm, respectively. Lesions typically occupy one to three depth layers of our seven-layer reconstruction, indicating that most lesions have a vertical diameter of 0.5 to 2 cm.

## 3 APU-Net Model

### 3.1 Overall Structure

**Fig. 3:**
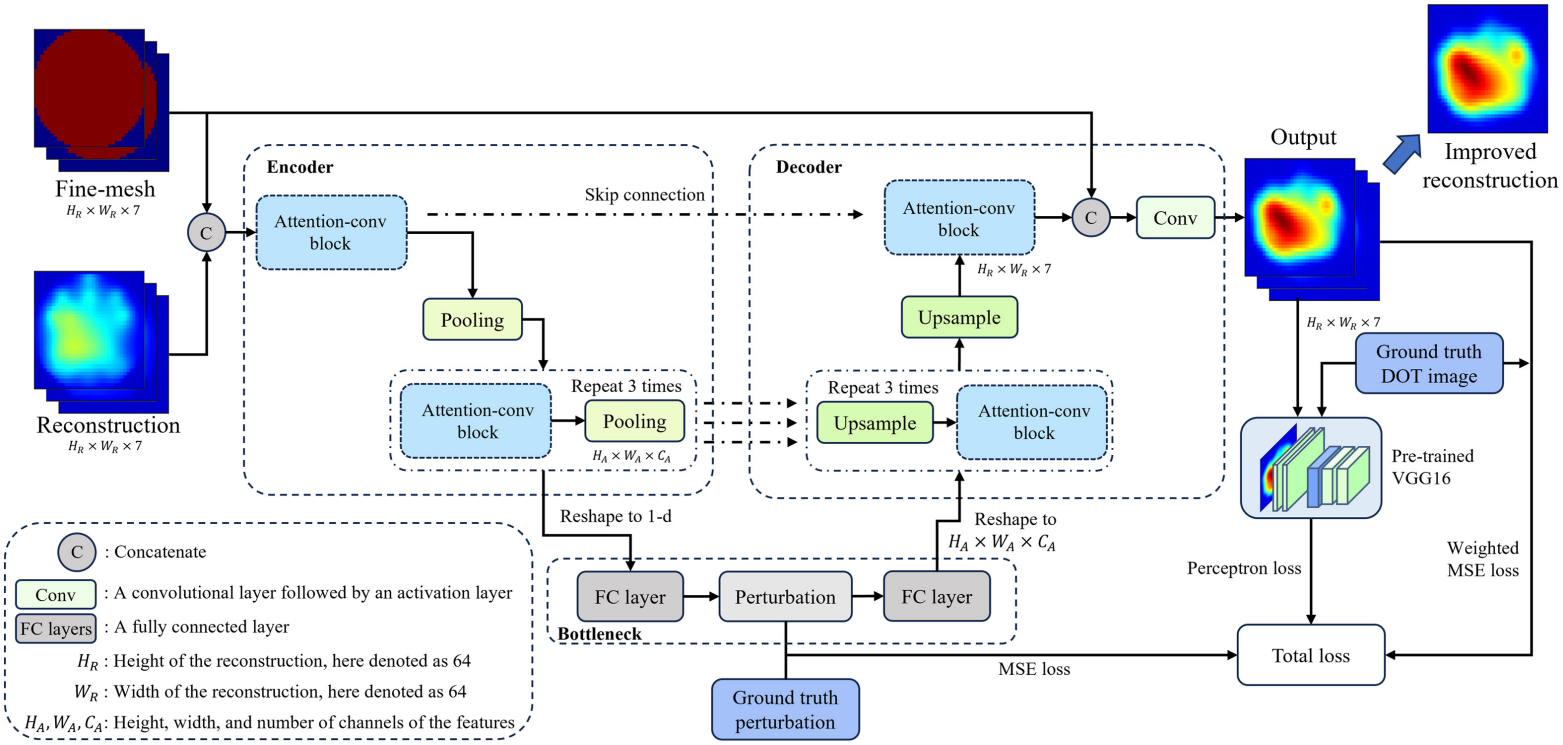
Structure of the APU-Net model. The encoder, up to the perturbation, functions as the solver for the photon diffusion equation, while the remaining neural network components serve as the solver for the inverse problem.

The overall network structure, depicted in Fig. 3, comprises two main components: an encoder, which solves the forward diffusion equation, and a decoder, which addresses the inverse problem, mapping perturbations to spatial absorption distributions.

In the encoder, the reconstruction DOT images and their corresponding fine-mesh regions serve as inputs, where the fine mesh delineates a refined grid in the spatial domain. By preserving the background as a coarser mesh grid, computational resources are concentrated on specified areas, enhancing the accuracy of the result. The concatenation of the reconstruction and fine mesh is then fed into an attention-convolution block, comprising a convolution layer followed by a CoT module, as elaborated in Sec. 3.3. Subsequently, a pooling layer compresses the features to a lower dimension. This module repeats four times, ultimately reshaping the output into a one-dimensional array.

In the bottleneck, multiple fully connected layers map the one-dimensional features to the perturbation, addressing the forward problem. The mean square error (MSE) loss between the bottleneck output and the perturbation is calculated as part of the final loss equation. The perturbation undergoes additional fully connected layers and is reshaped back to a three-dimensional form.

In the decoder, we begin by concatenating features from the encoder side as a skip connection, strategically employed to prevent overfitting. Then, four attention-conv blocks are utilized to decode these features into the reconstruction. We reinforce the spatial distribution emphasis by again concatenating the fine mesh with the features. Conclusively, two additional convolution layers are applied to acquire the enhanced reconstruction, solidifying the decoder’s role as the solver for the inverse problem in the diffusion equation.

### 3.2 CoT Attention Block

A DOT reconstruction, being a functional image, often lacks lesion detail due to low resolution, posing challenges for traditional deep learning models to accurately recognize targets and achieve satisfactory performance. To address this issue and improve the model’s focus on target areas within the DOT image, we introduced the CoT block as a self-attention module. The CoT module, designed to aggregate contextual information among input keys for guiding the learning of a dynamic attention matrix, demonstrates substantial potential in visual recognition. By linking neighboring information in keys to queries, it enables adaptive focus on the lesion and surrounding areas. This addition aids the model in understanding features more effectively and assigning attention to relevant areas.

**Fig. 4:**
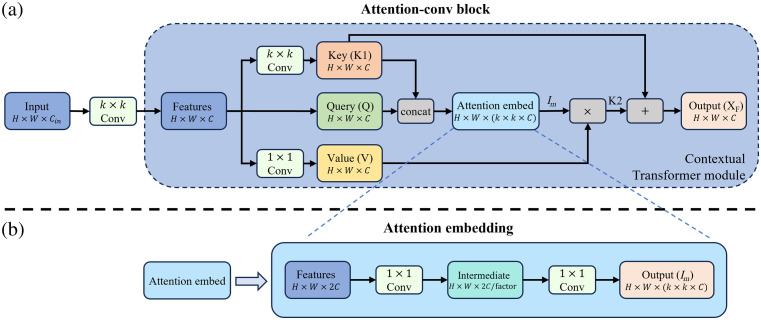
(a) Structure of the attention-conv block, where the “×” and “+” blocks denote the element-wise multiplication and addition operations, respectively. (b) Detailed architecture of the attention embedding module.

The CoT module’s structure, as outlined in Fig. 4(a), involves transforming a 3D feature map X (with dimensions H×W×C) into keys K=X, queries Q=X, and values $V=XW_{v}$, where $W_{v}$ is the embedding matrix, achieved through a 1×1 convolution. Departing from the traditional method, which uses 1×1 convolution to encode the keys, the CoT module utilizes k×k convolution over the neighbor keys within the k×k spatial grid. The learned keys, denoted as the mined static context $K_{1}$, take the shape of H×W×C. The module then embeds attention by concatenating $K_{1}$ and the Q then processing them with attention embedding $A_{e}$ which includes two consecutive 1×1 convolutions with a rectified linear unit activation after the first convolution:

$$I_{m}=A_{e}[K_{1},Q].$$ (3)

Here, unlike the isolated pairwise convolution, the CoT module combines the query with the key and the surrounding area at each location. Subsequently, the dynamic contextual representation of inputs $K_{2}$ is calculated as

$$K_{2}=V*\mathrm{softmax}(I_{m}),$$ (4)

where * stands for element-wise multiplication. Finally, the CoT module returns the fusion of the static context $K_{1}$ and the dynamic context $K_{2}$ as the refined feature map $X_{F}$ as^33^

$$X_{F}=\text{attention}(Q,K,V|X)=K_{1}+K_{2}=V*\text{softmax}(A_{R}[{\mathrm{conv}}_{k\times k}(K),Q]).$$ (5)

### 3.3 Loss Function and Training Schemes

To refine the model’s focus on lesions within the image, we employed a weighted MSE loss $L_{i}$ for reconstruction, expressed as

$$L_{i}(w)={\left\|a{\left(\theta (w|\delta \mu_{a},m_{f})-\delta \mu_{\left(a,g\right)}\right)}_{\text{target}}+b{\left(\theta (w|\delta \mu_{a},m_{f})-\delta \mu_{\left(a,g\right)}\right)}_{\text{back}}\right\|}_{2},$$ (6)

where θ represents the APU-Net, $\delta \mu_{a}$ and $m_{f}$ denote the reconstructed DOT images and corresponding fine mesh, respectively, and $\delta \mu_{\left(a,g\right)}$ signifies the ground truth. Finally, a and b represent the weights in the weighted MSE loss for the target area and background, respectively. $L_{i}$ adjusts weights to prioritize lesion areas, augmenting inclusion weights while reducing background weights.

In addition, to measure the semantic difference between the enhanced reconstruction and the ground truth, we utilized a pre-trained VGG16^41^ model to extract feature domain loss $L_{f}$ as the perceptron loss^42^

$$L_{f}(w)={\left\|F_{\mathrm{VGG}}(\theta (w|\delta \mu_{a},m_{f}))-F_{\mathrm{VGG}}(\delta \mu_{\left(a,g\right)})\right\|}_{2},$$ (7)

where $F_{\mathrm{VGG}}$ signifies the features of the pre-trained VGG16.

The MSE loss $L_{p}$ between the bottleneck output and the perturbation, as mentioned in Sec. 3.1, is calculated as

$$L_{p}(w)={\left\|\theta_{*}(w_{*}|\delta \mu_{a},m_{f})-U_{sc}\right\|}_{2},$$ (8)

where $w_{*}$ and $\theta_{*}$ represent the encoder portion up to the perturbation in APU-Net and $U_{\mathrm{sc}}$ represents the perturbation.

The overall loss combines perturbation and reconstruction aspects, defined as

$$\text{Loss}(w)=\alpha L_{p}(w)+\beta L_{i}(w)+\gamma L_{p}(w).$$ (9)

Here, α, β, and γ denote the weights for each loss, optimized during training.

In accordance with Sec. 2.2, we initially trained the model exclusively on multiple-target simulations, employing a learning rate of 0.0001 over 200 epochs. To manage the learning rate decay, a threshold of 0.01 was established, triggering adjustment if substantial loss drops were not observed within a specified epoch range. Subsequently, we conducted fine-tuning using single-target simulations and phantom data to reflect typical clinic scenarios. This phase employed a learning rate of 0.00005, consistent with the previous weight decay, and spanned 200 epochs. Following the training and fine-tuning, we determined the coefficients for loss calculation to be α=5, β=1, γ=0.01, a=0.98, and b=0.02, ensuring a balanced consideration of different components within the loss function from the grid search.

In addition, we applied various data augmentation techniques. These included adding random Gaussian noise to the original DOT reconstruction, rotating images randomly within a range of −45 to 45 deg, and applying random affine transformations. The affine transformations encompassed random variations in the rotation, translation, and scaling.

### 3.4 Evaluation Metrics

We assessed the performance of our model by measuring its effectiveness in removing artifacts and improving target contrast in different depth layers. To evaluate artifact contrast, we introduced the metric $C_{\mathrm{arti}}$, defined as the ratio of the maximum hemoglobin concentration within the artifact region to the maximum hemoglobin concentration within the lesion region

$$C_{\mathrm{arti}}=\frac{\max({\mathrm{hemo}}_{\mathrm{arti}})}{\max({\mathrm{hemo}}_{\mathrm{lesion}})}.$$ (10)

A lower value of $C_{\mathrm{arti}}$ indicates better artifact removal.

For depth contrast, we calculated the hemoglobin contrast among different depth layers. Specifically, we defined $C_{12}$ as the ratio of the maximum hemoglobin concentrations between the second and first depth layers, and $C_{13}$ as the ratio between the third and first layers. Unlike the artifact contrast, for $C_{12}$ and $C_{13}$, a value closer to one suggests that the hemoglobin contrast is consistent across layers, which is desirable.

## 4 Results

### 4.1 Test Results on Simulation Dataset

**Fig. 5:**
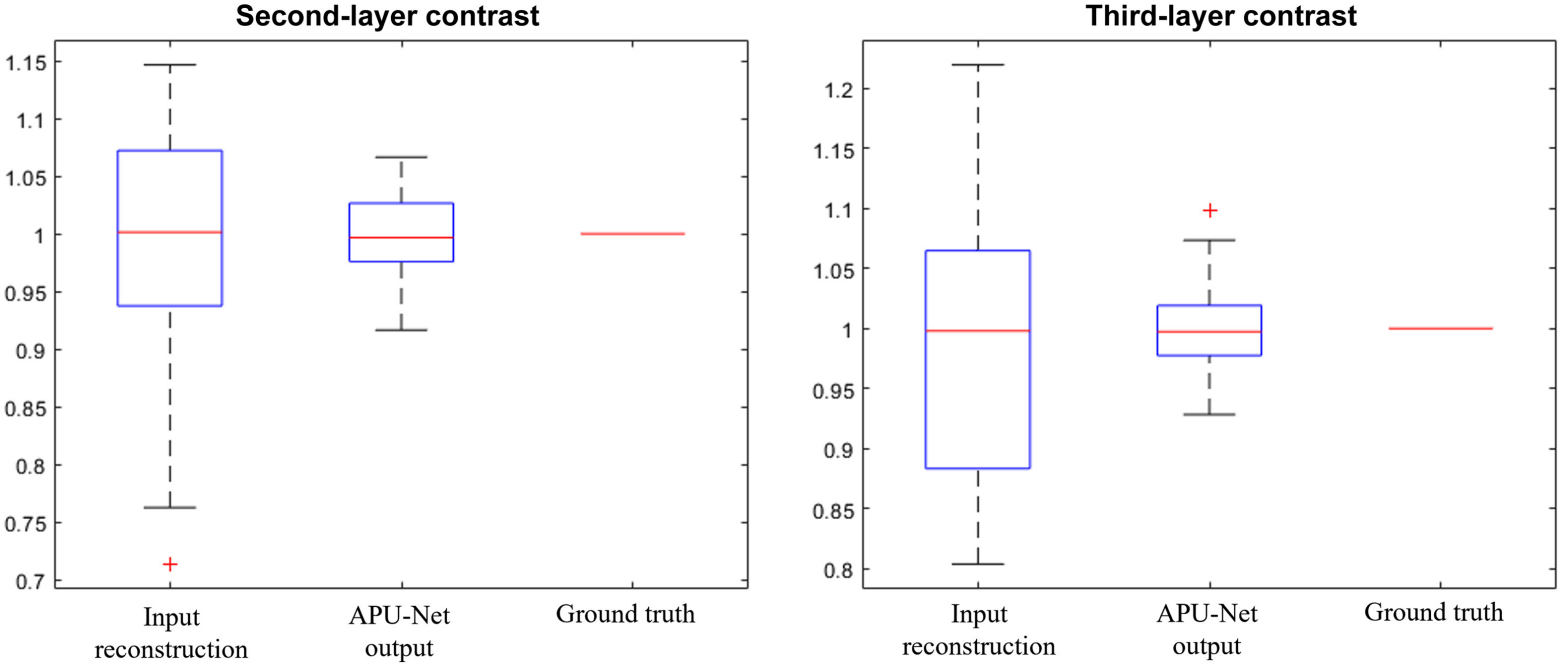
Comparative depth contrasts among the input reconstruction, the output of the model, and the ground truth.

We first evaluated the model’s performance using a separate simulation dataset. Figure 5 presents a boxplot comparing the input, output, and ground truth results, providing a detailed overview of the performance. In this dataset, which includes 1647 simulated cases, our APU-net successfully improved the depth contrast for both layers.

For the depth contrast between the first and second layers ($C_{12}$), our model increased the contrast from an initial average of 0.9926  (±0.1048) to 0.9989  (±0.0353). Similarly, for the depth contrast between the first and third depth layers ($C_{13}$), the model improved the contrast from 0.9836  (±0.1004) to 0.9997  (±0.0335). More importantly, the APU-Net significantly reduced the variance of the contrast.

### 4.2 Artifact Removal on Clinical Dataset

**Fig. 6:**
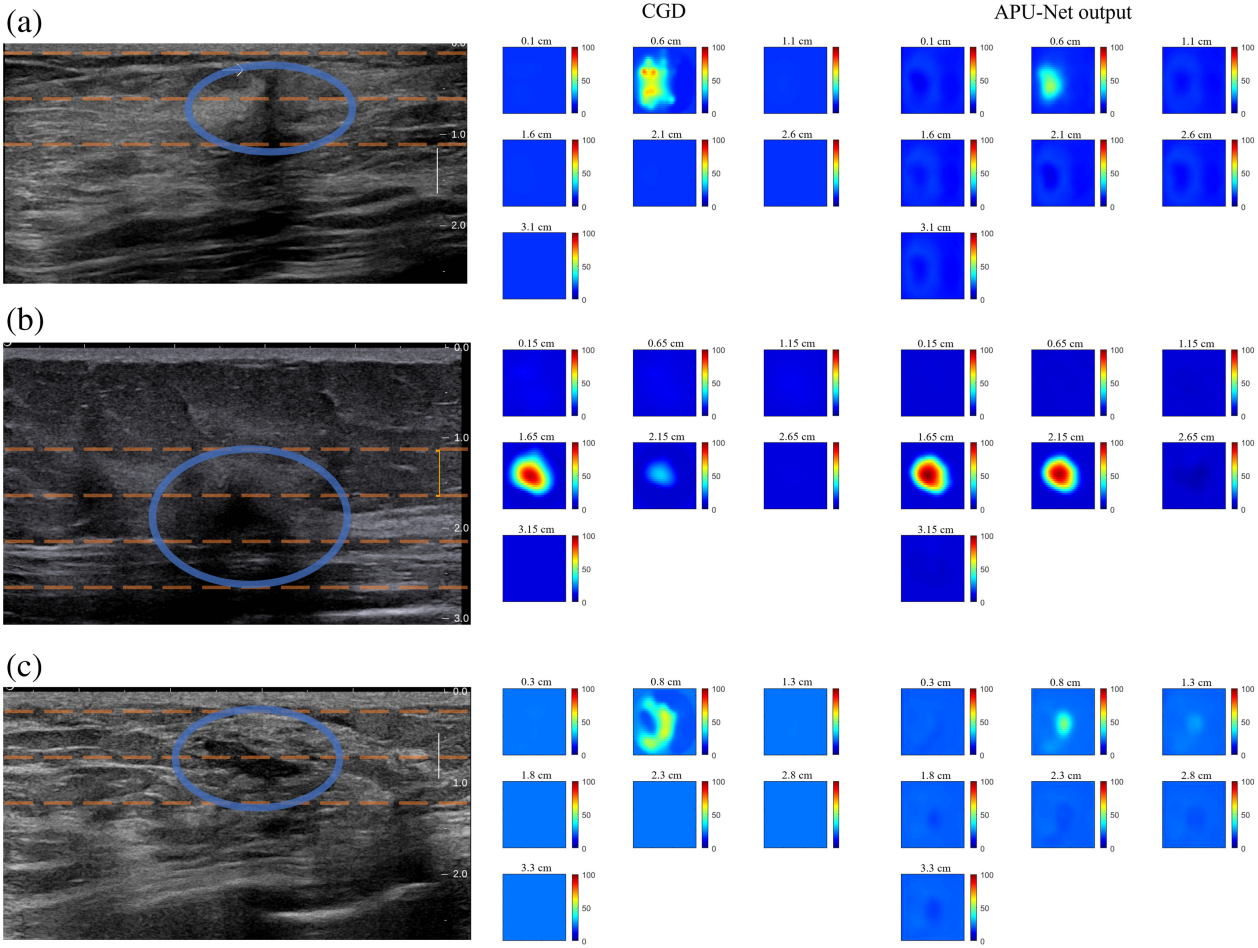
Examples of low-quality DOT reconstructions and corresponding corrected DOT images from the clinical dataset, with blue ovals indicating the locations of lesions. (a) A shallow malignancy with a source pattern obscuring the lesion’s top. (b) A deeper malignancy with shadow effects. (c) A benign lesion with artifacts attributed to tissue heterogeneity.

We then assess the model’s generalization to clinical datasets. We leverage examples of clinical DOT hemoglobin maps to showcase the model’s efficacy in artifact removal. Notably, the hemoglobin maps are derived from the absorption distribution at four wavelengths.^34^ Figure 6 presents three instances of low-quality reconstructions, accompanied by US images highlighting the targets on the left side, which include several dashed orange lines indicating the different depth layers of the lesion, along with one line representing the upper depth layer and one representing the lower depth layer.

In Fig. 6(a), we observe a malignant case at a shallow depth of 0.6 cm, where the lesion’s upper portion is obscured by sources near the probe surface. The model’s output on the right presents a more refined target at the center, with all source artifacts effectively removed. Figure 6(b) illustrates another malignant case, characterized by shadow effects stemming from intense absorption in the top layer. Here, our APU-Net model successfully restores the target’s absorption, aligning closely with the first layer’s shape and value, indicative of a favorable depth profile. Figure 6(c) showcases a benign case with artifacts caused by the heterogeneous background tissue. Here, the model adeptly identifies the lesion at the center while eliminating surrounding artifact-like regions, albeit exhibiting a lower maximum hemoglobin. Next, we compare the artifact contrasts of the original reconstructions and the outputs from the model.

**Fig. 7:**
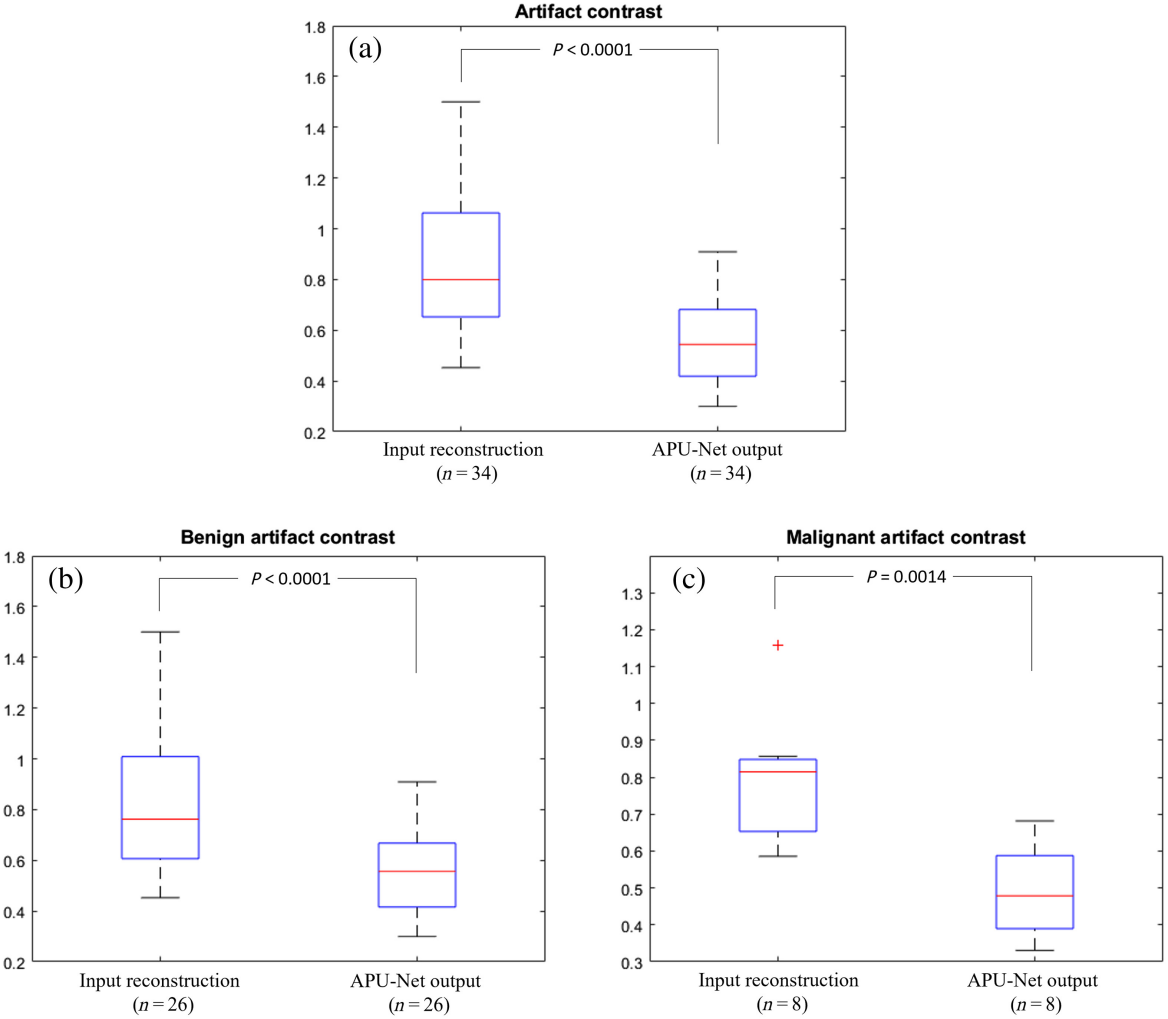
Comparison of artifact contrasts between the input reconstruction and the output of the model. (a) Artifact contrast for all cases. (b) Artifact contrast for the benign group. (c) Artifact contrast for the malignant group.

Figure 7(a), a boxplot of the inputs and outputs, shows the statistical details of the images. Based on 34 patients with artifacts caused by shallow targets or tissue heterogeneity, the model reduced the artifact contrast from 0.8448  (±0.2888) to 0.5580  (±0.1678). Figures 7(b) and 7(c) break down the artifact contrast by benign and malignant groups. For the benign group, the APU-Net decreased the artifact contrast from 0.8691  (±0.2901) to 0.5787  (±0.1755). In the malignant group, the artifact contrast was reduced from 0.7971  (±0.1785) to 0.4907  (±0.1263). These results clearly demonstrate the effectiveness of our model in reducing artifact contrast across different types of lesions.

These examples underscore the model’s seamless transition from simulation and phantom studies to clinical scenarios, demonstrating its robust performance. Subsequently, we provide further statistical analysis of the model’s efficacy on clinical data.

### 4.3 Statistics on Clinical Dataset

**Fig. 8:**
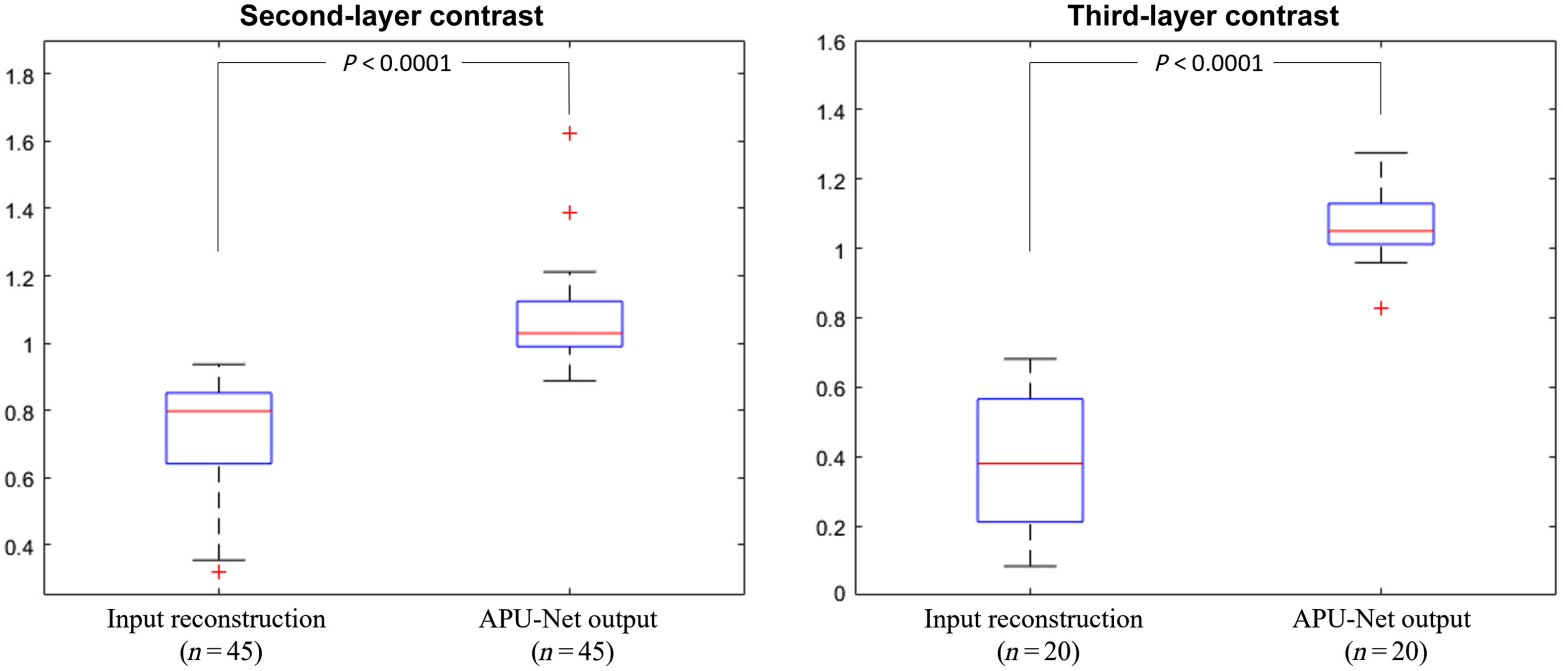
Contrast of hemoglobin between the second and first layers, as well as between the third and first layers.

To elucidate the model’s efficacy in enhancing depth profiles within clinical settings, we conducted a comprehensive analysis based on data collected from 83 patients. Among these patients, 45 had DOT reconstructions with more than two layers, while 20 had DOT reconstructions with three layers. In Fig. 8, we depict the maximum hemoglobin contrast as introduced in Sec. 3.3. After excluding six cases in the second-layer calculation and three cases in the third-layer calculation due to exceptionally low hemoglobin levels, our APU-Net model demonstrates notable improvement in contrast for deeper layers. Specifically, the contrast for the second layer $C_{12}$ increased from 0.7273  (±0.1650) to 1.0688  (±0.1379), while for the third layer $C_{13}$, it improved from 0.3811  (±0.1941) to 1.0611  (±0.1045).

**Fig. 9:**
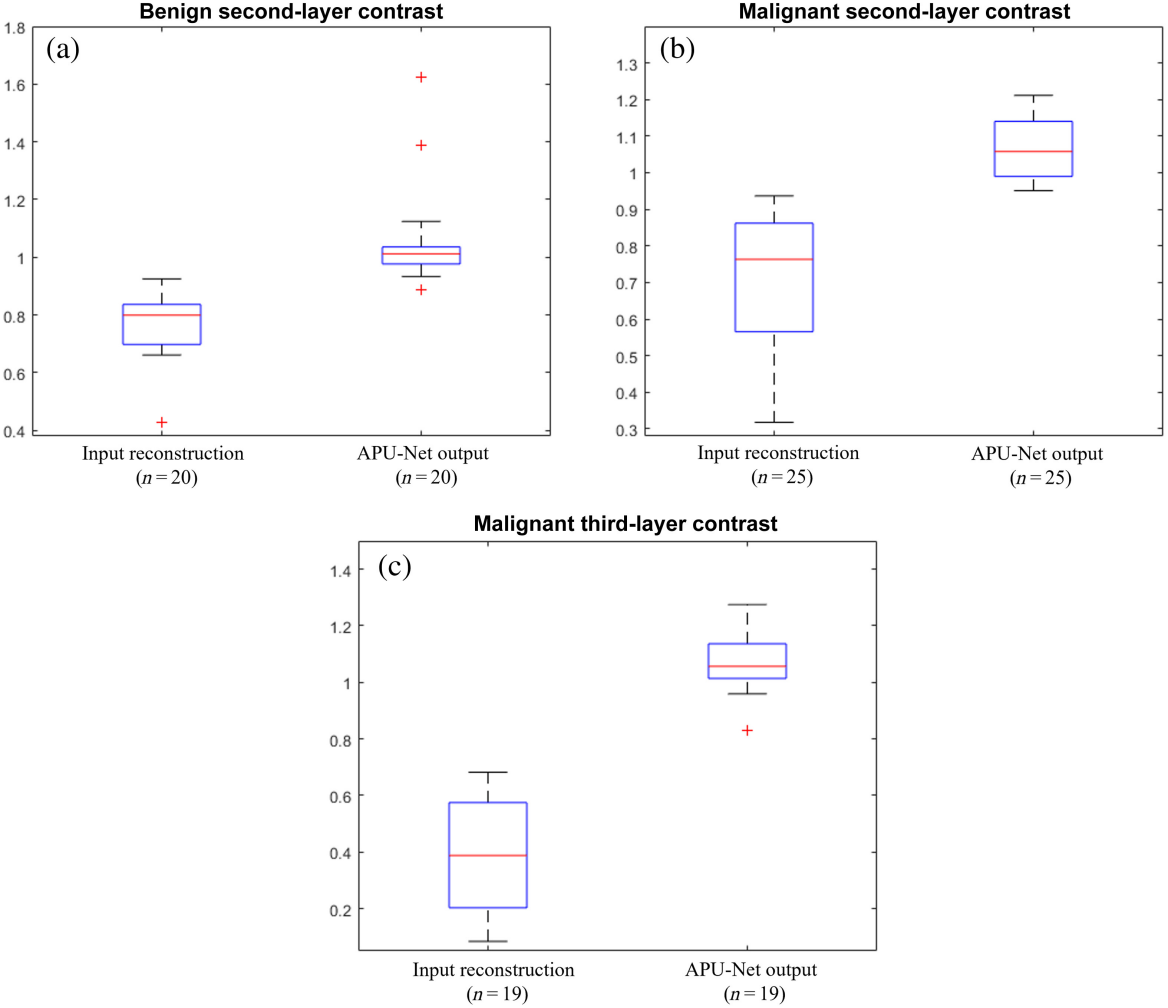
Depth contrast subgroup study. (a) Second-layer contrast for the benign group. (b) Second-layer contrast for the malignant group. (c) Third-layer contrast for the malignant group.

We then examined the depth contrast, separated into benign and malignant groups, as shown in Fig. 9. We observed significant differences in all subgroups (second-layer benign, second-layer malignant, and third-layer malignant), except for the third-layer contrast in the benign group, where only one patient’s data were available, making statistical analysis difficult. In the second layer, the depth contrast improved from 0.7634  (±0.1225) to 1.0724  (±0.1972) for the benign group and from 0.7020  (±0.1881) to 1.0663  (±0.0800) for the malignant group. For the third-layer contrast in the malignant group, the APU-Net increased the contrast from 0.3892  (±0.1987) to 1.0665  (±0.1063), indicating a significant improvement.

**Fig. 10:**
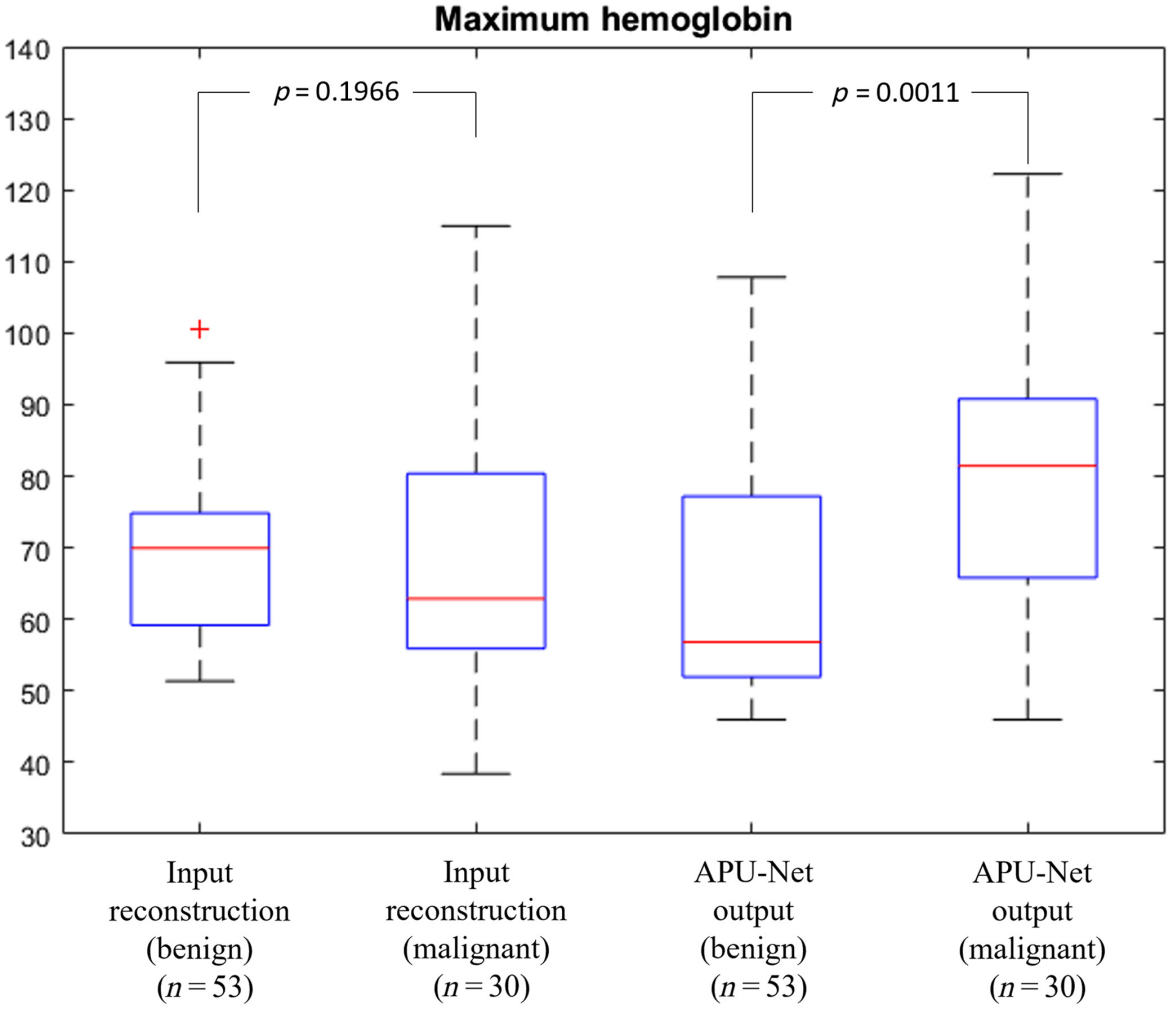
Maximum hemoglobin levels in benign and malignant groups, categorized by input reconstruction and APU-Net output.

We computed the maximum hemoglobin values, categorized by benign and malignant cases. For benign cases, the average output value is 64.24±18.83  μM, which is lower than the input average value of 69.84±12.56  μM. For the malignant cases, the averaged outputs are 82.70±20.67  μM, which is higher than the input hemoglobin of 75.73±21.57  μM, with a similar variance. There is no statistical significance between the input and output of the benign and malignant subgroups, respectively, which is expected since the goal of the study is to reduce image artifacts and improve the lesion depth profile. Furthermore, our analysis revealed that our APU-Net model enhanced the differentiation between benign and malignant groups, as illustrated in Fig. 10. This finding underscores the potential of our model to enhance diagnostic accuracy in future studies.

### 4.4 Ablation Study

Ablation studies were conducted to evaluate the impact of key design components in APU-Net on synthesis performance. We focused on the effectiveness of the attention module, by removing it to measure the enhancements facilitated by the attention blocks. To assess the impact of employing attention modules in the neural net, we evaluated the artifact contrast along with the depth profiles of the second and third layers in the DOT reconstruction.

**Fig. 11:**
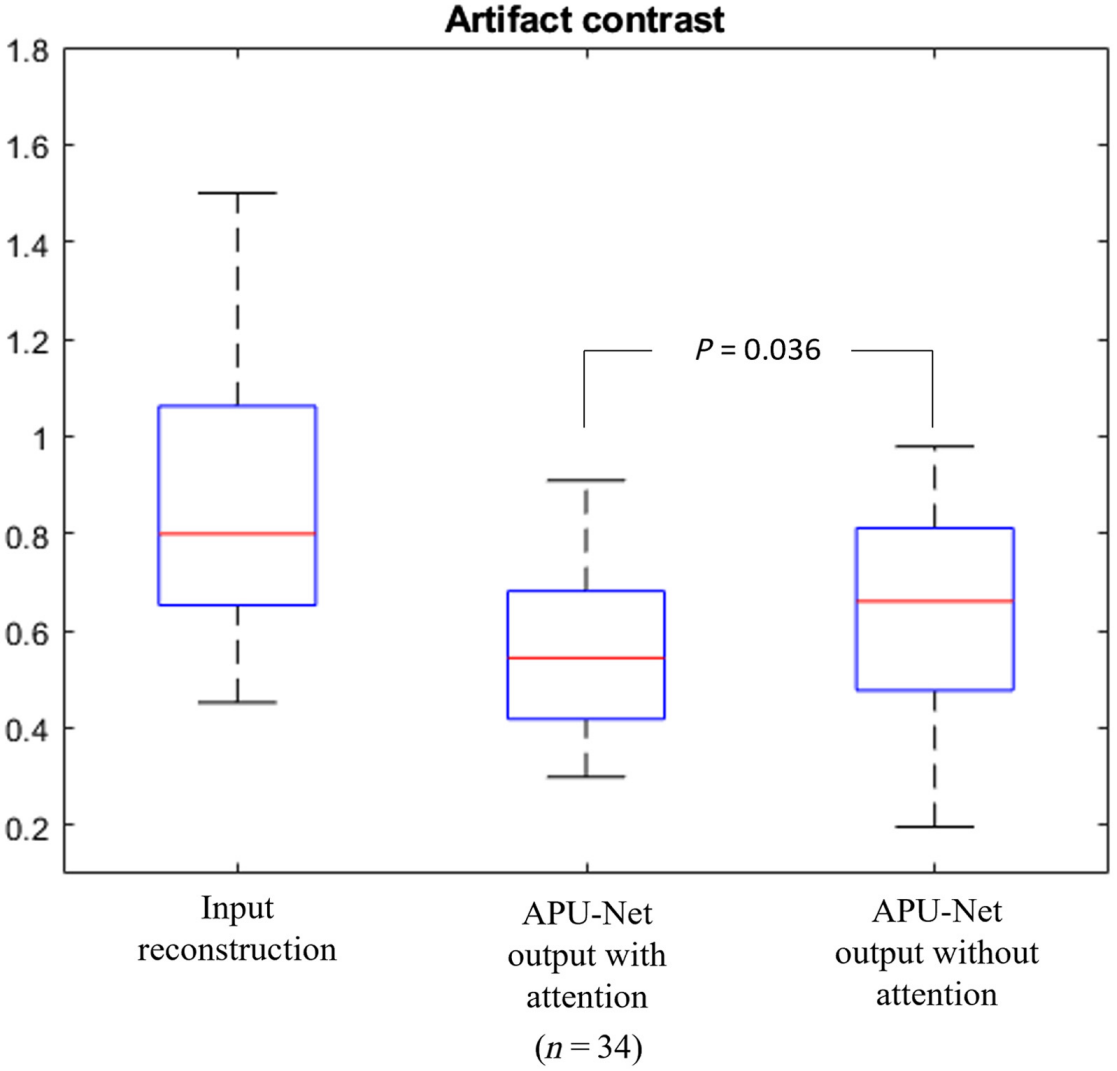
Artifact contrast for ablation study.

Figure 11 presents the results for artifact contrast. We observed a significant improvement when using APU-Net with the attention module than without it. APU-Net achieved an artifact contrast of 0.5580  (±0.1678), whereas the configuration without the attention module had an artifact contrast of 0.6530  (±0.1964).

**Fig. 12:**
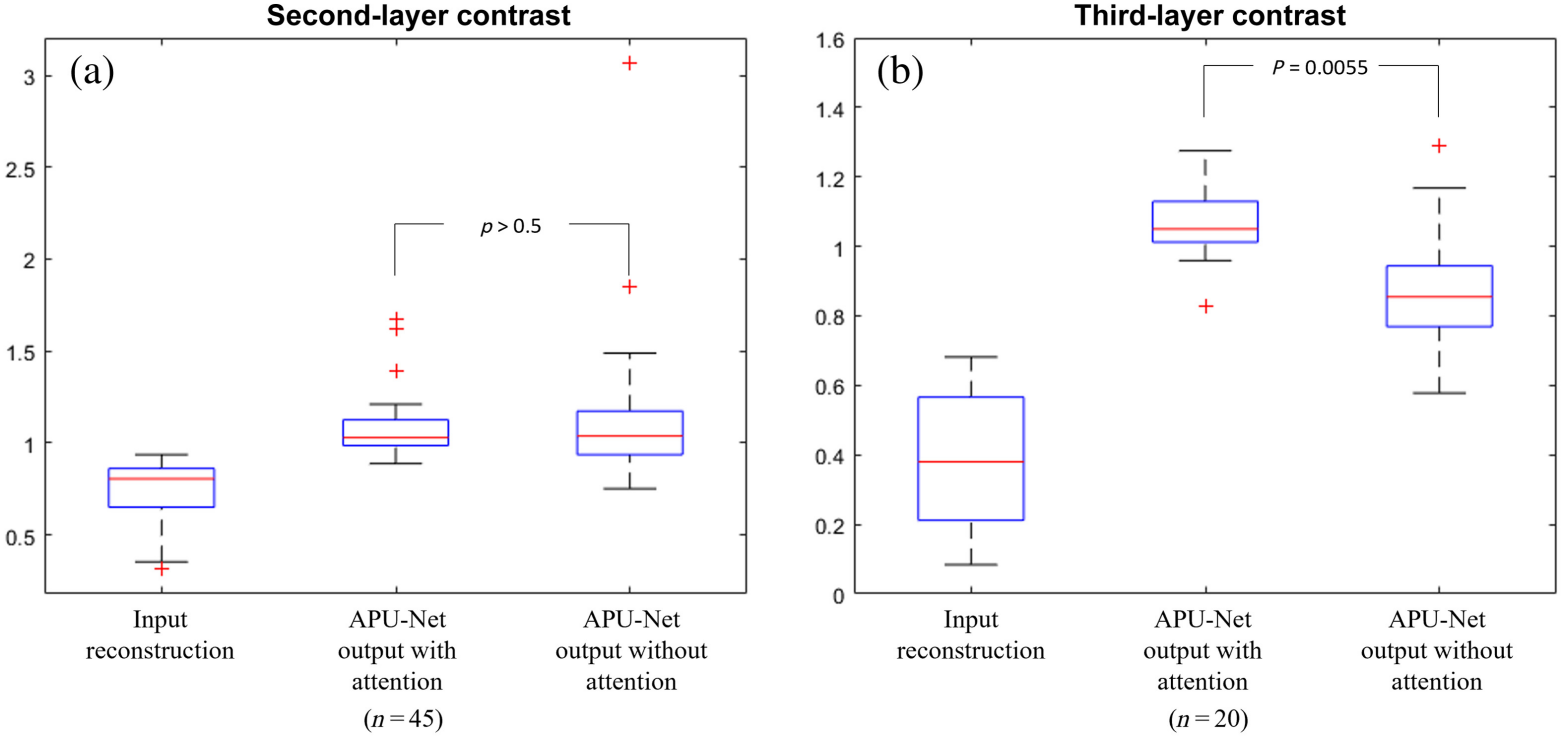
Deeper layer contrast for ablation study. (a) Second-layer contrast. (b) Third-layer contrast.

Figure 12 illustrates the contrasts of deeper layers versus the first lesion layer. Removing the attention modules from the model resulted in a second-layer contrast of 1.1169±0.3998 and a third-layer contrast of 0.8976±0.1826. While the model without the attention module showed a similar mean contrast compared with the current model, it also demonstrated larger variances either in the second or the third layer. In addition, the exclusion of the attention module during training led to ∼32% and 23% reductions in hemoglobin value for benign and malignant cases, respectively, emphasizing the critical role of attention modules in enhancing the model’s spatial distribution awareness and, consequently, preserving accurate values in the enhanced images.

## 5 Discussion and Conclusion

In this paper, we introduced an APU-Net model designed to enhance the quality of DOT reconstructions, effectively mitigating artifacts, improving depth profiles, and improving contrast in DOT images. The architecture of our model incorporates a U-Net structure augmented with a CoT attention module, followed by convolutional layers. The extraction of the bottleneck as the perturbation empowers the model to serve as a solver for both the forward diffusion equation and the inverse problem. Our training strategy, coupled with diverse target assignments in the simulation and phantoms, significantly enhances the model’s generalization to real-world clinical scenarios.

Transitioning to clinical datasets, our framework demonstrated robust generalization, successfully removing artifacts and improving image quality. This adaptability from simulation to clinical settings underscores its potential clinical utility in improving diagnostic accuracy. Statistical analyses further validate the efficacy of our approach, revealing significant improvements in artifact removal and depth profile contrast.

However, despite the promising performance of our model on low-quality clinical DOT reconstructions, there is room for improvement. The maximum hemoglobin values play a crucial role in our DOT study, as they can be important for downstream tasks such as differentiating between benign and malignant lesions. Although our model was not designed to perform this function, the analysis revealed that our APU-Net model enhanced the differentiation between benign and malignant groups. This finding underscores the potential of our model to enhance diagnostic accuracy in future studies.

In conclusion, while our model shows promising performance in enhancing DOT reconstructions, ongoing refinement and validation efforts are necessary to optimize its clinical utility and ensure its effective use in diverse patient populations.

## Data Availability

Associated code is uploaded to GitHub (https://github.com/OpticalUltrasoundImaging/DOT_filtering). Data are available from the corresponding author upon reasonable request.
